# Toyoko Kids: A case series on the psychosocial backgrounds of eight runaway girls transported to emergency care in Tokyo

**DOI:** 10.1111/ped.70248

**Published:** 2025-11-05

**Authors:** Shunichiro Nakamura, Tetsuto Baba, Natsuko Tokita, Chikako Shimizu, Ryo Yamamoto, Satoshi Narumi

**Affiliations:** ^1^ Department of Pediatrics Keio University School of Medicine Tokyo Japan; ^2^ Department of Emergency and Critical Care Medicine Keio University School of Medicine Tokyo Japan; ^3^ Department of Emergency and Critical Care Medicine National Hospital Organization Kumamoto Medical Center Kumamoto Japan

**Keywords:** child abuse, overdose, runaway teens, self‐harm, single parent

## Abstract

**Aim:**

Runaway behavior, defined as leaving home without parental permission and not returning, is associated with adverse educational, physical, and mental health outcomes. In Japan, since around 2018, a group of runaway adolescents known as “Toyoko Kids” has emerged in Tokyo's Kabukicho area, reportedly engaging in substance use, violence, and prostitution. This study aimed to examine the psychosocial characteristics of Toyoko Kids who were transported to emergency care due to impaired consciousness from drug overdose or alcohol intoxication.

**Methods:**

We conducted a retrospective chart review of eight female adolescents under 18 years old, transported from Kabukicho to Keio University Hospital between June 2022 and August 2023. Inclusion criteria included having run away for at least two nights (or one night for those under 15) and being transported by a third party. A multidisciplinary team assessed psychosocial risks using a Bio‐Psycho‐Social model. Data on consciousness level, pregnancy, substance use, self‐harm, psychiatric history, family structure, school attendance, abuse, and child welfare involvement were analyzed and compared with national statistics.

**Results:**

All subjects were female, aged 12–16. The most common reason for transport was impaired consciousness due to overdose, primarily involving over‐the‐counter medications. Self‐harm was confirmed in 86% and abuse in 67% of cases. Although several had prior psychiatric or welfare intervention, support was often temporary and insufficient. Most came from single‐parent households, and over half had a history of school absenteeism.

**Conclusions:**

This study identified severe psychosocial vulnerabilities among Toyoko Kids. Findings highlight gaps in mental health and child protection systems.

## BACKGROUND

Runaway behavior refers to children leaving home without parental permission and not returning. The Office of Juvenile Justice and Delinquency Prevention defines a runaway as a child under 15 years old who does not return home for at least one night, or a child aged 15 or older who does not return for at least two nights.^1^, ^2^ In the United States, 5–8% of adolescents experienced runaway behavior annually, and the behavior was associated with adverse educational, physical, and mental health outcomes.^1^, ^3^, ^4^ About 75% of these youths fail to complete high school, and they face increased risks of sexually transmitted infections, unintended pregnancies, and suicide attempts.^3^, ^4^, ^5^, ^6^ Notably, the frequency of suicide attempts by American youth experiencing both homelessness and runaways was reported to be 33%, clearly higher than the 2% of youth with stable housing.^3^

In Japan, while the National Police Agency publishes statistics on missing persons, specific data on runaways are not available. Since around 2018, runaway youths, known as “Toyoko Kids,” have become an increasing social concern. Hundreds of teenagers from various regions within approximately 100 km of Tokyo are living on the streets or in temporary accommodations, particularly in Kabukicho, one of Japan's most infamous nightlife districts.^7^ This area is notorious for incidents involving underage drinking, drug abuse, violence, and prostitution. Despite frequent media coverage, there is a notable lack of public health research on Toyoko Kids.^7^

These urban phenomena, such as the clustering of unsupervised youth in nightlife districts, reflect a convergence of structural challenges—including family dysfunction, poverty, and inadequate social safety nets—that may heighten the vulnerability of adolescents. This concentration appears to reflect broader systemic issues—such as housing instability, family dysfunction, and limited accessibility of child welfare support. Such conditions may constitute an important social backdrop for understanding the psychosocial risks explored in this study.

Prior research has shown that early attachment disruptions and unstable caregiving environments contribute to emotional and behavioral dysregulation in adolescence.^8^, ^9^ In the absence of protective factors—such as consistent caregiving or supportive adult relationships—adolescents may resort to maladaptive coping strategies, including self‐harming behaviors.^10^ Grounded in these theoretical foundations, we adopted the Bio‐Psycho‐Social (BPS) model to assess risk factors across biological, psychological, and social domains.

We examined the psychosocial characteristics of eight adolescent girls, referred to as “Toyoko Kids,” who received emergency care after being found in Kabukicho. Assessments were guided by the BPS model, which provided a structured clinical framework for identifying vulnerabilities across multiple domains. To our knowledge, this is among the first public health studies to systematically investigate the psychosocial profiles of Toyoko Kids using such an approach.

## METHODS

We specifically focused on runaway adolescents who had been emergently transported to a tertiary care hospital. Unlike community‐based surveys or school‐reported cases, emergency transports allow for the direct clinical observation of severe risk behaviors such as overdose and impaired consciousness. This subgroup, though not representative of all runaways, provides valuable insight into the intersection of medical urgency and psychosocial vulnerability.

This study is a cross‐sectional analysis of Toyoko Kids who were emergently transported to Keio University Hospital from June 2022 to August 2023. Subjects were included in the study if they met the following three criteria: (i) youth under age 18 years who were transported from Kabukicho in Shinjuku Ward, (ii) who had not returned home for more than two nights (or more than one night for those under age 15 years) without the caregiver's permission, and (iii) the emergency call was made by someone other than family (e.g., friends or passersby). Cases due to traffic accidents or accidental injuries were excluded.

Clinical interviews and physical examinations were conducted by pediatricians for all cases. Although no structured questionnaire or standardized checklist was used, assessments were guided by the BPS model and conducted through direct interviews. This approach was chosen because adolescents with trauma or high‐risk behaviors may be reluctant to disclose sensitive information through self‐administered questionnaires due to fear, shame, or mistrust, as reported in previous studies.^11^, ^12^ Under the supervision of the first author—a pediatrician and board‐certified specialist in child and adolescent mental health—attending clinicians assessed key psychosocial risk factors according to their clinical roles, and relevant findings were documented in medical records.

Based on this framework, specific roles and responsibilities were allocated across clinical domains (Table 1). In the biological domain, emergency physicians and emergency department nurses primarily assessed the level of consciousness, diagnoses, pregnancy status, substances ingested, and treatments provided. In the psychological domain, pediatricians and psychiatrists evaluated self‐harming behaviors, psychiatric symptoms, substance use history, and suicidal ideation. In the social domain, pediatricians and medical social workers gathered information on family structure (e.g., both parents, single parent, or others), current school attendance status, history of abuse, involvement with child protection services, and experiences of sexual exploitation. Information on who requested the emergency call and the distance from home to Kabukicho was obtained from emergency and medical records. Pediatricians and psychiatrists jointly conducted interviews with both the children and their caregivers, while pediatricians worked with medical social workers to share relevant information with child protection services and coordinate follow‐up support. Discharge planning—including decisions such as home return or custody—was conducted through multidisciplinary discussions among pediatricians, emergency physicians, psychiatrists, nurses, and social workers. All data were obtained as part of routine clinical care, not through research‐specific interviews. Interviews were conducted in private rooms within the emergency department, without the presence of caregivers, to ensure a safe and confidential environment. Pediatricians and psychiatrists took care not to pressure the adolescents to disclose information, and responded empathetically to any signs of emotional distress.

**TABLE 1:** Multidisciplinary roles in Bio‐Psycho‐Social Care for Toyoko kids.

	Emergency physicians	ED nurses	Pediatricians	Psychiatrists	MSWs
Biological domain					
Vital signs assessment	●	●	●		
GCS assessment	●				
General blood test	●				
Toxicology screening	●				
Pregnancy test	●				
Acute interventions (e.g., IV infusion, intubation)	●				
Psychological domain					
Self‐harm history	●	●	●	●	
Suicidal ideation assessment				●	
Psychiatric treatment history			●	●	●
Substance use history	●		●	●	
Social domain					
Family structure assessment			●	●	●
School attendance check			●		
CPS involvement history			●		●
Abuse history assessment			●		
Sexual exploitation inquiry			●		
Notification to child protection services			●		●
Coordination with community services (e.g. return home, police custody)	●	●	●	●	●

*Note*: Each “●” indicates the professional responsible for assessing the corresponding item within the BPS framework.

Abbreviations: ED, emergency department; IV, intravenous; MSW, medical social worker.

The definition of abuse was based on the third revised edition of the Clinical Guidelines for Child Abuse Management published by the Japanese Pediatric Society^13^ Physical abuse included acts such as beating, slapping, squeezing, and grabbing the child. Psychological abuse was defined as actions that frightened the child or denied them emotional responses. Sexual abuse included forced sexual acts or being forced to watch sexual acts. Neglect was defined as failing to provide necessary supervision for the child's safety or emotional care essential for development.

Characteristics of the study subjects were compared with those of the Japanese general population. Reference data were obtained from official reports by the Ministry of Health, Labor and Welfare and the Ministry of Education, Culture, Sports, Science and Technology, as well as from previous studies on adverse childhood experiences, self‐harm behaviors, and drug use among adolescents.^14^, ^15^, ^16^, ^17^, ^18^ To account for missing values, only complete cases were used for proportion calculations. Variables were categorized into biological, psychological, and social domains based on clinical relevance, following the BPS model. For categorical variables with small sample sizes, Fisher's exact test was used instead of chi‐square test to ensure appropriate statistical validity. To account for multiple comparisons (*n* = 6), a Bonferroni correction was applied, and the significance threshold was adjusted to 0.0083 (0.05/6). Effect sizes were calculated as odds ratios with 95% confidence intervals for group comparisons in Table 3, to indicate the magnitude and clinical relevance of the findings.

The study was approved by the Ethics Committee of Keio University School of Medicine (Approval number: 2018212). The study was conducted as a retrospective analysis using existing data, and therefore, individual informed consent was not obtained. Instead, the research adhered to an opt‐out approach, in accordance with ethical guidelines with study information posted on the Keio University Hospital website and hospital notice. All data were anonymized at the time of extraction, and no personally identifiable information was used in the analysis.

## RESULTS

During the study period, nine runaway adolescents were emergently transported to Keio University Hospital, eight of whom were from Kabukicho. The eight unrelated female subjects were aged between 12 and 16 years (median, 15 years). The most common reason for transportation was impaired consciousness due to drug overdose, observed in five cases—four involving over‐the‐counter (OTC) medications and one involving prescription medication. Two cases involved acute alcohol intoxication, and one case involved both OTC drug overdose and alcohol intoxication. Clinical characteristics were organized into biological, psychological, and social domains in accordance with the BPS model and were summarized in Table 2.

**TABLE 2:** Characteristics of the study subjects.

	*n*/*N*	%^a^
Biological domain		
Gender		
Girl	8/8	100
Age (year)		
~12	1	12.5
13–15	5	62.5
16–17	2/8	25.0
Reason for visit		
Overdose of OTC meds	4	50.0
Overdose of prescribed meds	1	12.5
Alcohol intoxication	2	25.0
OTC and Alcohol	1/8	12.5
Consciousness level upon arrival		
GCS 13–15	6	75.0
9–12	0	0.0
3–8	2/8	25.0
Medical procedures and hospitalization		
Blood test only	3	37.5
Blood test and IV infusion	4	50.0
Intubation and transfer to HCU	1/8	12.5
Pregnancy	1/8	12.5
Psychological domain		
Substance use		
Alcohol	5	62.5
Smoking	3	37.5
Illegal drug use	1/8	12.5
Self‐harm	6/7	85.7
Suicidal ideation	2/8	25.0
Psychiatric treatment history		
Outpatient	1	12.5
Hospitalization	2/8	25.0
Social domain		
Caregiver(s)		
Single mother	5	62.5
Single father	1	12.5
Father and mother	2/8	25.0
Siblings		
Present	6/7	85.7
Distance from home to Kabukicho (km)		
0–30	3	37.5
31–60	4	50.0
61–90	1/8	12.5
Caller for emergency response		
Friend	2	25.0
Building Security Guard	1	12.5
Police officer	5/8	62.5
School absenteeism		
Not attending	4	50.0
Drop out	1	12.5
Attending^b^	3/8	37.5
History of abuse		
Physical	3	33.3
Sexual	1	16.6
Neglect	1/6	16.6
History of child welfare		
Protective custody	3	42.9
Intervention		
Intervention (details unknown)	3/7	42.9
Sexual exploitation	2/7	28.6
Discharge destination		
Under police custody	1	12.5
Home	7/8	87.5

Abbreviations: IV, intravenous; OTC, over‐the‐counter.

^a^ Percentages were calculated based on complete cases (*n*/*N*).

^b^ One of the three attending students was enrolled in an online high school.

Upon hospital arrival, three patients were fully conscious [Glasgow Coma Scale (GCS) E4V5M6], three had mild impairment of consciousness (GCS E3‐4V3‐4M6), and two had severe impairment of consciousness (GCS E1V1M1–5). Three patients were discharged after physical examination and blood tests. Four patients received intravenous fluid therapy in addition to physical examination and blood tests. One patient required intensive care with endotracheal intubation and ventilation. Blood tests revealed no illegal drugs except for alcohol. One of them was pregnant.

Alcohol use was reported in five subjects, smoking in three, and marijuana use in one. Wrist self‐harm was seen in five subjects, four of which were recent. Suicidal ideation was recognized in two subjects. History of psychiatric consultation was noted in three subjects: two had been hospitalized for treatment, and one had received outpatient treatment.

In all eight cases, the overdose or alcohol intoxication was not their first such experience. Among them, six had a documented history of prior emergency transport due to similar incidents. Although the frequency and timing of these events were not consistently recorded, these findings suggest that the risk behaviors observed in this cohort were not isolated episodes but part of a broader pattern of recurrent self‐harm or maladaptive coping.

Of the eight subjects, six were from single‐parent families: five were living with their mothers, and one with her father. The distance from Kabukicho to their homes ranged from 8 to 83 km (median 37 km). Of the seven subjects from whom sibling information was collected, six had siblings: four were the eldest of two, one was the eldest of three, and one was the youngest of four.

One subject had dropped out of high school. Among the remaining seven, all were enrolled in either junior high or high school: two had attended school within a week of the emergency transportation, one was attending a correspondence school, and four were not attending school. A history of abuse was confirmed in four of the six evaluated subjects, including physical abuse (*N* = 2), both physical and sexual abuse (*N* = 1), and emotional neglect (*N* = 1). Two of these subjects were experiencing abuse at the time of the emergency transportation. Among the eight participants included in the study, seven were asked about their past involvement with child protection services. Three reported having been placed under protective custody, and another three had a history of child protection intervention, although details were unknown. Two out of seven subjects disclosed experiences of being sexually exploited. Seven of the patients returned home, while one was placed under police protection.

The characteristics of Toyoko Kids were compared with those of the general Japanese population (Table 3). The frequency of single‐parent households among the study subjects was 75.0% (6 out of 8), markedly higher than that in the general population (7.7%).^14^ Five of the eight subjects (62.5%) had long‐term school absenteeism, which is approximately 15 times more frequent than in the general population (4.1%).^15^ Frequencies of history of physical abuse (50.0%), mental neglect (16.7%), and sexual abuse (16.7%) were higher than those in the general population (physical abuse 4.1%; mental neglect 16.5%; sexual abuse 6.9%).^16^

**TABLE 3:** Comparison with general population.

Variable	Study subjects	General population	*p*‐value^a^	Odds ratio (95% CI)^b^
Single‐parent household	75.0%	7.7%^14^	6.4 × 10^−6^	36.0 (7.3–178.2)
Long‐term absenteeism	62.5%	4.1%^15^	8.1 × 10^−6^	39.0 (9.3–163.1)
Physical abuse history	50.0%	4.1%^16^	2.1 × 10^−4^	23.4 (5.9–93.5)
Emotional neglect	16.7%	16.5%^16^	1.0	1.0 (0.1–8.7)
Sexual abuse	16.7%	6.9%^16^	0.35	2.7 (0.3–23.1)
Self‐harm	85.7%	12%^17^	2.2 × 10^−5^	44.0 (5.3–365.5)

*Note*: A Bonferroni correction was applied for multiple comparisons (*α* = 0.0083).

^a^ *p* values were calculated using Fisher's exact test due to small sample size.

^b^ Odds ratios and 95% CIs were calculated using study‐group variability; general‐population rates were treated as fixed benchmarks from official reports.

Self‐harm was observed in 85.7%, a frequency seven times compared with Japanese adolescent girls.^17^ Overdose on OTC medications was noted in 62.5%, a notably higher frequency than Japanese high school students (1.6%) who reported OTC drug misuse.^18^

## DISCUSSION

This study reports on eight adolescent girls, known as “Toyoko Kids,” who were transported to emergency care after drug overdose or alcohol intoxication in Kabukicho, Tokyo. A key feature of the study is the structured psychosocial assessment based on the BPS model. The findings revealed a complex interplay of physical issues (e.g., acute intoxication, pregnancy), psychological issues (e.g., self‐harm, suicidal ideation), and social adversities (e.g., single‐parent households, school absenteeism, abuse).

All participants were female, and most had been exposed to multiple adversities, such as family separation, abuse, and neglect. Similar demographic and psychosocial patterns have been documented in prior research on runaway and overdose behaviors among youth in the United States and South Korea.^19^, ^20^, ^21^, ^22^ For example, 5–8% of adolescents in the U.S. and approximately 3% of middle and high school students in South Korea have experienced running away. These behaviors are not unique to highly industrialized nations but rather represent broader global challenges related to youth vulnerability. Prior research in high‐income countries has shown that family dysfunction is a common underlying factor in runaway behavior, self‐harm, and overdose among adolescents.^23^, ^24^ Divorce, as one example of family disruption, is associated with domestic violence in 20–30% of cases, exposing children to abuse and neglect.^25^, ^26^ After divorce, caregivers often face emotional exhaustion and financial stress, which may further impair caregiving capacity and increase the risk of maltreatment.^27^ These adverse environments can lead to academic difficulties, school refusal, interpersonal problems, self‐harm, substance use, sexual exploitation, and teenage pregnancy.^28^, ^29^, ^30^, ^31^, ^32^ Moreover, young people who experience such adversities may face an increased risk of reproducing dysfunctional family dynamics in the future.^33^, ^34^ In this study, six out of eight participants were raised in single‐parent households, highlighting the urgent need for early and sustained support for adolescents with such backgrounds.

Despite six of the seven participants having a history of child protection service involvement, they continued to engage in high‐risk behaviors such as self‐harm and overdose. These behaviors are often rooted in mental health conditions such as depression, anxiety disorders, and trauma‐related symptoms, requiring appropriate psychiatric assessment and treatment.^35^ However, only three of the eight participants had a history of psychiatric care, indicating gaps in service provision. Pediatricians must assess psychosocial risks and promptly report suspected abuse to child protection services. To address underlying conditions such as depression, anxiety disorders, and neurodevelopmental disorders, referral to mental health professionals is often necessary. Clinical coordination by pediatricians can help bridge the fragmented support systems across health care, education, and welfare sectors. Preventing the escalation of risk behaviors requires early identification of high‐risk families through preschools and schools. It also calls for continuous involvement by public health nurses or school counselors, as well as improved collaboration between primary care pediatricians and educational or welfare institutions.^36^, ^37^

One notable finding of the present study was the high rate of overdose of OTC medications, observed in 62.5% of participants. National surveys in Japan have also reported a rise in psychiatric symptoms associated with inappropriate OTC drug use among youth. These medications are often perceived as low‐risk, leading to underestimation of their toxicity, and may be misused for overdose or in combination with other drugs, resulting in serious health consequences.^38^ Although some age restrictions exist in Japan, current regulatory measures are insufficient, especially regarding online sales and unauthorized reselling, which allow youth to repeatedly access these substances. A policy review is urgently needed. In addition to regulatory reform, school‐ and community‐based preventive education programs are essential. Systems for early detection and intervention in self‐harming behaviors linked to substance use should be further strengthened.

This study has several limitations. First, as a descriptive case series from a single tertiary care facility, the findings may not be generalizable to other regions of Japan. However, similar groups of vulnerable adolescents have been reported in other major cities such as Osaka, Nagoya, and Fukuoka. Multicenter studies are needed to explore regional similarities and differences. Second, the small sample size limits statistical power. Future studies involving larger populations should allow for quantitative analyses of associations between psychosocial risk factors and high‐risk behaviors, as well as the identification of predictive factors through multivariate analysis. Third, due to the cross‐sectional design, the study could not examine the strength of associations, temporal changes, or the effects of interventions. Longitudinal and interventional research will be necessary to develop practical support models. Finally, given the sensitivity of topics such as substance use, exploitation, and family adversity, the possibility of under‐reporting or response bias cannot be excluded.

In summary, we analyzed the psychosocial vulnerabilities of adolescent girls transported from Kabukicho for emergency medical care using the BPS model. The findings offer practical implications for the fields of child welfare, psychiatry, and public health. Future clinical and policy efforts should focus on strengthening early detection via medical contact, fostering inter‐system collaboration, and implementing regionally tailored, multi‐agency support frameworks.

## AUTHOR CONTRIBUTIONS

ShN conceptualized and designed the study, collected and curated the data, performed statistical analysis, drafted the initial manuscript, critically revised the manuscript, and supervised the entire research process. TB, NT, CS, and RY contributed to data collection. SaN conceptualized and designed the study, critically revised the manuscript, and supervised the overall research process. All authors read and approved the final version of the manuscript and agree to be accountable for all aspects of the work.

## FUNDING INFORMATION

This study received no specific funding.

## CONFLICT OF INTEREST STATEMENT

ShN serves as Chair of the Research Committee of the Japanese Society of Psychosomatic Pediatrics, which is unrelated to the present study. All other authors declare no conflicts of interest.

## Data Availability

The data that support the findings of this study are openly available in figshare at https://figshare.com/s/c4feb391fe3a5652417f.
